# Exploring multi-level system factors facilitating educator training and implementation of evidence-based practices (EBP): a study protocol

**DOI:** 10.1186/s13012-017-0698-1

**Published:** 2018-01-08

**Authors:** Aubyn C. Stahmer, Jessica Suhrheinrich, Patricia L. Schetter, Elizabeth McGee Hassrick

**Affiliations:** 1 University of California, Davis MIND Institute, 2825 50th St, Sacramento, CA 95819 USA; 2 College of Education, San Diego State University, 5500 Campanile Dr., San Diego, CA 92182 USA; 3 Department of Psychiatry and Behavioral Sciences, University of California, Davis, Sacramento, USA; 4 Center of Excellence in Developmental Disabilities, University of California, Davis, Sacramento, USA; 5 Child and Adolescent Services Research Center, San Diego, USA; 6 Life Course Outcomes Research Program at AJ Drexel Autism Institute, Drexel University, 3020 Market St, St 560, Philadelphia, PA19104 USA

**Keywords:** Multi-level system factors, Special education, Autism, Evidence-based practices, Implementation, Teacher training

## Abstract

**Background:**

This study examines how system-wide (i.e., region, district, and school) mechanisms such as leadership support, training requirements, structure, collaboration, and education affect the use of evidence-based practices (EBPs) in schools and how this affects the outcomes for students with autism spectrum disorder (ASD). Despite growing evidence for the positive effects of EBPs for ASD, these practices are not consistently or effectively used in schools. Although special education programs are mandated to use EBPs, there are very few evidence-based methods for selecting, implementing, and sustaining EBPs. Research focuses primarily on teacher training, without attention to contextual factors (e.g., implementation climate, attitudes toward EBPs, resource allocation, and social networks) that may impact outcomes. Using an implementation science framework, this project will prospectively examine relations between system-wide factors and teachers’ use of EBPs and student education outcomes.

**Methods/design:**

Survey data will be collected from approximately 85 regional special education directors, 170 regional program specialists, 265 district special education directors, 265 behavior specialists, 925 school principals, 3538 special education teachers, and 2700 paraprofessionals. Administrative data for the students with ASD served by participating teachers will be examined. A total of 79 regional-, district-, and school-level personnel will also participate in social network interviews. Mixed methods, including surveys, administrative data, and observational checklists, will be used to gather in-depth information about system-wide malleable factors that relate to positive teacher implementation of EBPs and student outcomes. Multi-level modeling will be used to assess system-wide malleable factors related to EBP implementation which will be linked to the trainer, teacher, and student outcomes and examined based on moderators (e.g., district size, Special Education Local Plan Area structure, teachers’ ASD experience). Finally, a dynamic social network approach will be used to map EBP-related connectivity across all levels of the system for selected regions. Dynamic network analysis will be used to gauge the degree to which and ways that EBP trainings, resources, and interventions are shared (or not shared) among school staff.

**Discussion:**

Results are expected to inform the development of system-wide interventions to improve the school-based implementation of EBPs for students with ASD.

## Background

The Centers for Disease Control (CDC) estimates that 1 in 68 children have an autism spectrum disorder (ASD; [1]). Long-term outcomes for this population are poor [2–5], and the annual cost in the USA is estimated to be $268 billion [6]. The service system accessed most often for school-age children with ASD is education (Brookman-Frazee, Baker-Ericzen, Stahmer, Mandell, Haine, and Hough [7]). The number of children with ASD served by schools has grown fivefold from 93,000 in 2000 to 455,000 in 2011. The education system is responsible for targeting a wide range of needs that interfere with a child’s ability to benefit from general education including improving learning skills such as attention and engagement and core symptoms of ASD.

The federal legislation known as “Every Child Succeeds,” passed in 2015 [8], and the Individuals with Disabilities Education Act [9], both specify that the practices used in schools must be those supported by scientifically based evidence. Additionally, specific evidence-based practices (EBPs) for ASD have been identified for school personnel [10–13]. However, research indicates a large gap between research and school practices [14] with EBP for ASD not being routinely used in schools [15, 16]. Similar to gaps in education, generally in ASD, teachers and other school-based providers can learn to use EBPs when trained by experts [17] but do not typically incorporate EBPs into school-based programs over the long term [18–20]. This is problematic because children with ASD show significant gains when they receive services implemented with high fidelity and when schools do not use interventions with proven efficacy; the courts can require them to provide costly alternatives, such as increased one-to-one intervention or placement in a private school [21, 22].

Scaling up interventions across multiple schools, districts and statewide present an additional challenge. Most state systems have a very limited capacity for scaling up interventions in ways that lead to meaningful improvements in outcomes for students [23]. Based on the research in mental health systems, it is clear that facilitation of effective EBP use requires leadership coordination and support at the school, district, and state levels; however, leaders at each of these levels have a very limited understanding of the factors beyond their immediate locale, such as system-wide policies and support, that may lead to gaps in the use of EBP in special education. While recent systematic reviews and meta-analyses of care coordination consistently report differences (of a moderate to medium effect size) in outcomes of mental health intervention and physical conditions management programs when collaborative practices within systems are compared to usual care [24–26], the “active ingredients” that allow for systematic implementation of coordinated care across systems remain obscure. There are critical gaps in evidence about the structure, function, and benefits of team coordination across multiple systems.

School-based service for special education involves a team of providers and administrators to account for the complexity of care needed for this population of students. For example, California has 1028 school districts divided into 122 regional consortiums for the purpose of providing for all special education service needs of children in the regions. These *Special Education Local Plan Areas* (SELPAs) work alongside school district staff to meet the children’s educational and mental health needs. They offer varying levels of support and structure from taking an active role in effective implementation of EBPs by providing autism specialists to support training and implementation to simply providing oversight for compliance. High-level administrators at the SELPA and school district levels are responsible for the provision of educational and associated programming, including staffing allocation, curricula, and use of resources. Administrators report that scientific evidence does not readily affect their programming decisions [27]. Administrators at both the district and SELPA level likely have a strong influence on the culture and climate related to the use of EBP as well as resource allocation for training, materials, and supervision in the use of new practices. Of course, principals play an important role in the leadership of an individual school site. However, only a small percentage of principals was special educators prior to their administrative roles and have limited professional training in special education [28]. Additionally, preliminary data indicate teachers and other high-level school district staff perceive special education directors, mid-level specialists, and teachers as key decision-makers across phases of implementation for ASD programs as compared to school site principals [29]. Teachers and paraprofessionals are front-line EBP users. They are the ones to adapt and modify practices and to determine the use. At each level of administration, resource allocation, implementation climate, education, experience with ASD, attitudes toward EBP, and collaboration have the potential to affect EBP use. Therefore, systems and processes at multiple levels must be considered for implementation in special education.

### Conceptual model

**Fig. 1:**
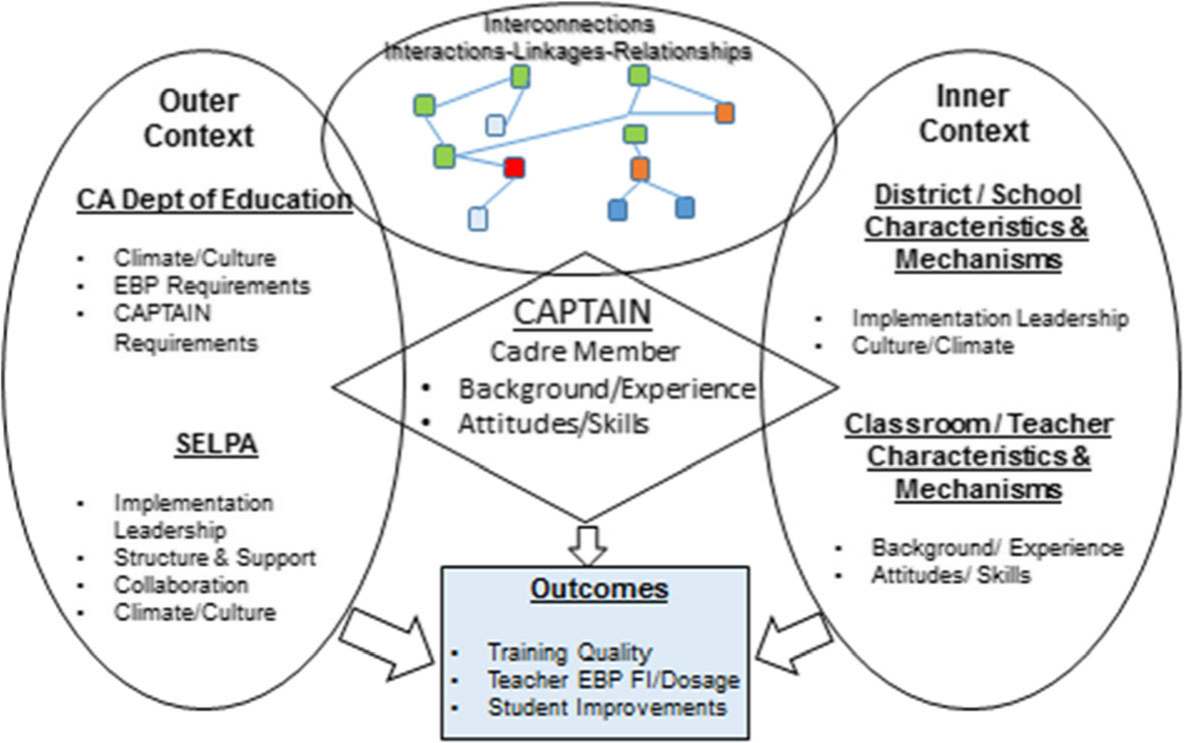
Applying the Exploration, Preparation, Implementation, and Sustainment (EPIS) conceptual model to ASD EBP in schools. This figure illustrates how we will use the Exploration, Preparation, Implementation, and Sustainment (EPIS) Implementation Model (Aarons, Hurlburt, and Horwitz 2011) to frame this exploratory study of facilitators and barriers to implementing statewide teacher training in EBP. This figure depicts the multi-level framework and indicates factors influencing implementation, including outer (e.g., state- and SELPA-level climate and culture, leadership and structure) and inner (e.g., leadership, teacher characteristics) contexts and social connections within and among system levels. We will use this framework to explore cross-context malleable factors and their potential influence on teacher training in EBP and student outcomes

There have been urgent calls for the development and testing of implementation interventions to facilitate successful uptake and sustained delivery of EBP in schools. Glisson and Williams [30] call for carefully designed, multi-level studies testing specific change mechanisms as they affect both leader- and provider-level factors; however, there is currently very limited research examining cross-level mechanisms linking specific implementation interventions to targeted changes in provider behaviors [31]. We apply the Exploration, Preparation, Implementation, and Sustainment (EPIS) model [32] (see Fig. 1) to frame the proposed exploratory study of facilitators and barriers to implementing statewide teacher training in EBP. EPIS was developed for a public child services context and integrates a multi-level framework to highlight factors influencing implementation, including outer (e.g., state and SELPA level climate and culture, leadership, and structure) and inner (e.g., leadership, teacher characteristics) contexts, and social connections within and among system levels. We will use this framework and a current implementation effort (CAPTAIN, see below) to explore *cross-context malleable factors* and their potential influence on teacher training in EBP and student outcomes.

Data from previous studies of EBP implementation outcomes from community effectiveness trials provide specific direction on promising factors related to EPIS that can be leveraged within growing, large-scale translation efforts. Several possible malleable factors including implementation leadership and climate, district-/SELPA-level resources and support, teacher attitudes toward EBP and teacher/paraprofessional skills, and social connections are associated with successful implementation [33, 34].

Implementation leadership can improve the climate for use of EBP [35]. Additionally, a clear relationship between organizational culture and climate and child-level outcomes has been identified in educational settings for ASD [36]. Positive implementation climate coupled with the use of support strategies (training availability, ongoing monitoring of performance, etc.) has been linked to better sustainment of innovation, improved child outcomes and decreased staff burnout [37], and predicted higher EBP fidelity [36]. Provider attitudes toward EBP have been linked to practice behavior [38, 39] and have been shown to predict use of EBP [32, 40, 41]. Finally, there is considerable evidence that team coordination, when done well, can change the outcomes [42]. Coordination impacts treatment quality [43] and goal attainment [44] for children with ASD. All of these factors may be important for EBP implementation in special education and may be points of intervention to improve outcomes.

### Current California implementation and scale up efforts

**Fig. 2:**
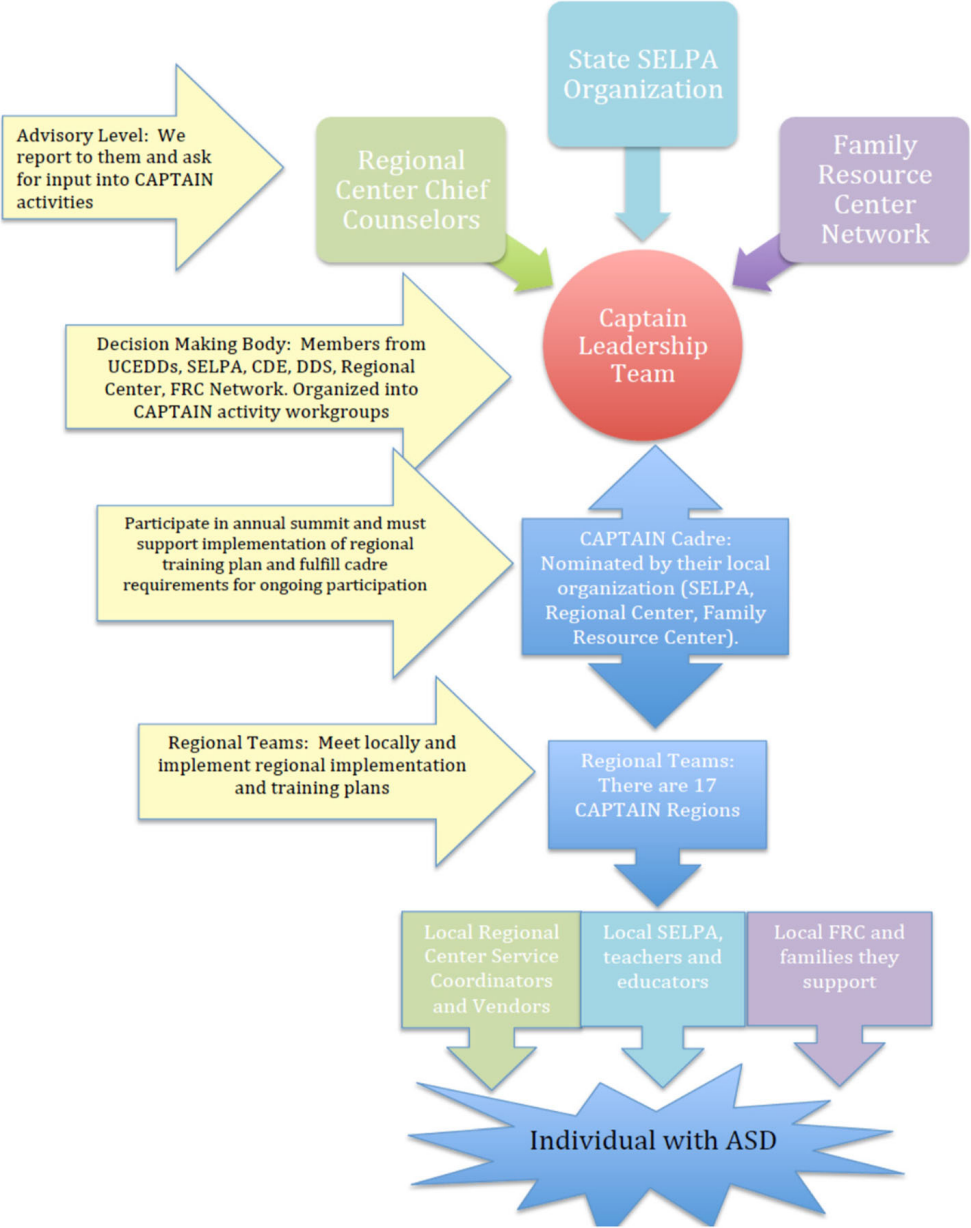
California Autism Professional Training and Information Network (CAPTAIN) organizational structure. Shows the organizational structure of the CAPTAIN network. A leadership team of cross-agency representatives (*red circle*) communicates directly with the California State SELPA Directors and guides the training and support efforts of the organization. Cadre members from each SELPA are nominated annually and are required to provide a certain amount of training to teachers in their areas each year and to attend an annual conference on EBP. Regional teams meet locally to implement training for local SELPA teachers and educators

The California Department of Education—Special Schools Division participated in a project sponsored by the National Professional Development Center for Autism Spectrum Disorders (NPDC-ASD, 2009). As part of their implementation plan, the state developed the *California Autism Professional Training and Information Network* (*CAPTAIN*), a collaboration of service providers from three statewide support agencies: Special Education Local Plan Areas (SELPAs), California Regional Centers (RCs), and Family Resource Centers (FRCs). The goal was to establish a training and technical assistance network for service providers with a focus on EBP for ASD. CAPTAIN was established in 2012 and currently has over 400 members. A majority of members (77%) are SELPA/school district personnel from across California. A leadership team of cross-agency representatives guides the training and support efforts of the organization (see Fig. 2 and http://www.captain.ca.gov for more information about CAPTAIN). CAPTAIN cadre members from each SELPA commit to provide a certain amount of training to teachers in their areas each year and to attend an annual conference on EBP. Preliminary data indicate successful outcomes in terms of increasing the frequency of training [45]; however, there is variability in the amount and types of training provided, and no data are available regarding teacher or student outcomes. Additional data are needed to understand system- and educator-level factors that lead to successful implementation to maximize CAPTAIN efforts. Targeted exploration of the statewide CAPTAIN model as a platform for professional development will contribute to the limited data on how system-level practices affect school-based intervention and student outcomes not only for students with ASD but for all served by special education programs.

### Research objectives (see Fig. 3)

1. Measure system-level factors that may relate to the teacher and student outcomes such as implementation climate and culture, communication and collaboration, and EBP readiness.
2. Measure the special education teacher outcomes (EBP training received, fidelity of implementation, dosage, and knowledge of EBP) and ASD student outcomes (time spent in the least restricted environment (LRE), disciplinary action, attendance, and IEP progress)
3. Measure potential moderation variables across three levels: SELPA/district (size, geography, structure), school (implementation leadership, attitudes toward EBP), and teacher (background, experience, attitudes toward EBP, climate)
4. Use innovative social network methodology to examine how collaboration across the multi-level system affects outcomes.
5. Assess moderation of system-level factors on CAPTAIN trainer outcomes (amount of time spent training, coaching, supporting trained staff), teacher outcomes (EBP fidelity of implementation, dosage, EBP knowledge) use, and student outcomes (LRE, IEP progress, discipline/behavior, attendance).

**Fig. 3:**
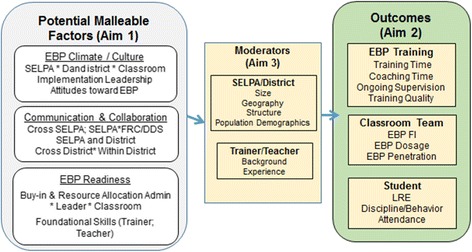
Factors, mechanisms, and outcomes. Illustrates the project aims, including the potential malleable factors identified in the literature, potential moderators of those factors, and proposed teacher training and student outcomes to be measured

## Methods/design

### Study design

This exploratory study will use mixed methods that include primary data collection from authentic educational settings at the SELPA, district, and school level and will access administrative student data. Components include a collection of multi-level survey data, student outcome administrative data, and in-depth interviews to conduct a social network analysis in a subset of programs.

### Participants and recruitment strategy

**Fig. 4:**
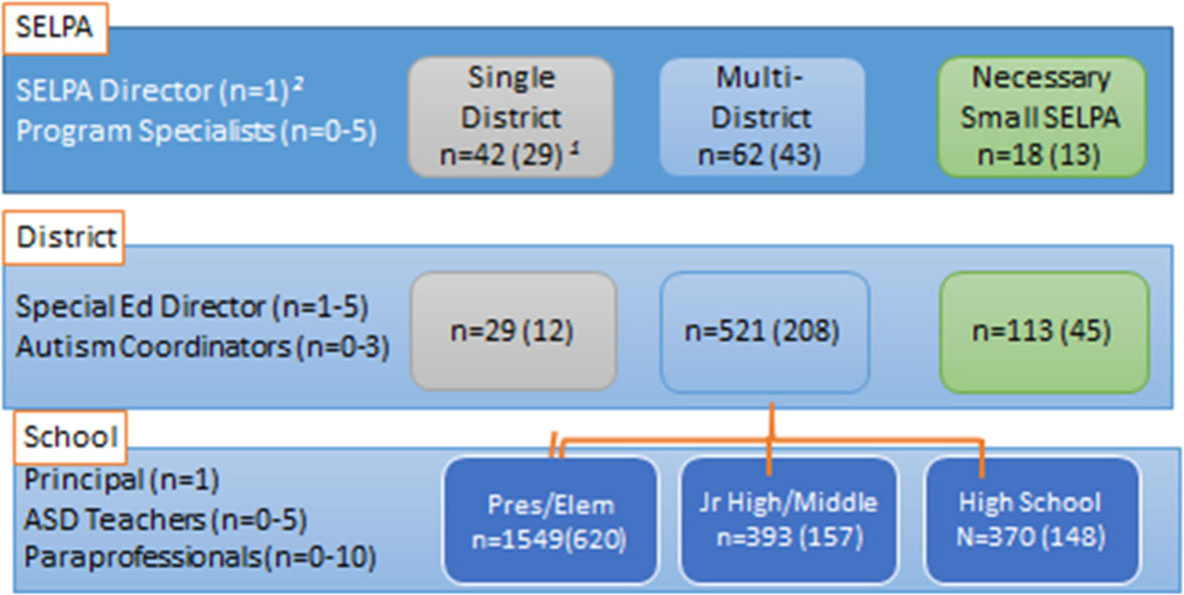
Estimated participation across levels. Illustrates the identified sample. At the SELPA level, our goal is 70% participation with representation from at least 30 of the 42 single district SELPAs, 13 of the 18 necessary small SELPAs (NSS; all multi-district), and 43 of the 62 multi-district SELPAs for a total of 85 SELPA directors surveyed. Participating SELPA directors in multi-district SELPAs will be asked to distribute study information to district directors of special education/student services. SELPA directors will invite their program specialists. We anticipate a 40% response rate at each of the subsequent levels. District special education directors will be asked to distribute study information to all of their autism/behavior coordinators and principals/site administrators at elementary, middle, and high schools (including special education schools and preschools), who will then distribute to each special education teachers and paraprofessional educators serving children with ASD within their district. There are on average 8.6 schools per district. Principals will then invite each special education teacher (including resource specialists) and paraprofessionals serving children with ASD. This will vary by district as well as by student enrollment; however, there are approximately 3.7 special education teachers and 5.8 paraprofessional educators per school across the state. CAPTAIN cadre member trainers will be included in their home SELPA/district and will also complete some additional information if they have a role as a trainer.^1^Estimated number of people invited (estimated number participating); ^2^Estimated range per SELPA/district/school

Our sample will include administrators and providers at multiple levels with data collected in a cascading format (see Fig. 4). We anticipate the participants will include SELPA directors (*n* = 85), SELPA program specialists (*n* = 170), district special education directors (*n* = 265), school principals (*n* = 925), autism specialists/coordinators (*n* = 265), special education teachers (*n* = 1375 participants), and paraprofessionals (*n* = 2700 participants). We anticipate a total of approximately 5700 participants. The CAPTAIN cadre member will be included in their home SELPA/district and will also complete the CAPTAIN survey and some additional information if they have a role as a trainer.

*Students*: Inclusionary criteria for students include (1) enrollment in a participating classroom and (2) a primary educational classification of ASD. De-identified data for students in a particular district will be provided by the SELPA/district directors to the research team. Data will not be linked to specific students.

*Survey data collection*. We will use a web-based platform for survey distribution, and it will be available for completion over a 3-month period. The estimated response rate is 70% for SELPA directors and 50% of distribution for all other populations [46]. SELPA directors will be contacted at their monthly state directors meeting and asked to complete the surveys. Directors will nominate all program specialists and special education directors from each district in their SELPA. Special education directors will give all autism/behavior coordinators and principals at elementary, K-12, middle, and high school campuses study information, and in turn, principals will provide information to their special education teachers and paraprofessionals serving children with ASD. CAPTAIN cadre members (trainers) will complete the surveys at an annual CAPTAIN conference, by phone or online. The research team will work with special education directors to distribute surveys and answer questions about the project. Weekly follow-up emails will be made to anyone who has not yet completed the survey to answer any questions and facilitate survey completion.

### Measures

We have chosen measures based on the literature indicating system-level malleable factors that affect implementation success. Table 1 lists the study measures and who will complete each measure. CAPTAIN cadre trainers will complete a set of measures, and student data will be obtained from appropriate state and district databases. Many of the implementation measures have multiple (slightly modified) versions for paraprofessionals, teachers, principals, and administrators.

**Table 1:** Study measures and timeline

Purpose (aim)	Construct	Measure/indicators	Service level					
			SELPA	District	School (princ)	Trainer	Teacher/para	Student
Malleable factors	Imp. climate	Implementation Climate Scale (ICS)	x	x	x	x	x	
	Org. culture	Organizational Social Context for schools	x	x	x	x	x	
	Imp. leadership	Implementation Leadership Scale	x	x	x	x	x	
	Attitudes	Evidence-based practice attitude scale (EPBAS) Implementation citizenship behavior	x	x	x	x	x	
	Resource allocation	ASD EBP Resource Assessment Tool (adapted from Program Sustainability Assessment Tool)	x	x	x			
	Social networks	Social network egocentric measures of advice providers (general and CAPTAIN) and network density	x	x	x	x	x	
Moderators	Program size	Number of students in district or SELPA	x	x				
	Proportion of students with ASD	Proportion of students with identified ASD served in the district	x	x				
	SELPA structure	Multi-district/single district/necessary small (NSS)	x					
	Poverty level	Proportion of students in district with free/reduced lunch	x	x	x			
	CAPTAIN participation	Years of participation with goals met						
	Primary discipline	Educational training and discipline			x	x	x	
	ASD experience	Years of experience working with children with autism			x	x	x	
Outcomes	Training quality	Training survey (self-created Likert scale) quality questions (*teacher/para report on trainer behavior*)					X	
	Training dosage	Training and confidence survey; dosage and type (didactic; coaching; supervision)				x	x	
	Implementation citizenship	Implementation Citizenship Behavior Scale (*trainer report on teacher behavior*)				x		
	EBP dosage	Training and confidence survey; report of use					x	
	EBP knowledge survey	Survey of Educators Knowledge and Value of Research-Based Practices for Students with Autism					x	
	EBP FI	NPDC checklists					x	
	LRE	Proportion of educational time in general education classroom; receipt of intensive individual services (Y/N); placement type (residential; separate school/class; regular classroom)						x
	Behavior	Number of days that include a behavior incident report, suspension or expulsion						x
	Attendance	Number of school days attended						x

#### Malleable factors

*Implementation and Attitudes.* The *Implementation Climate Scale* (ICS; [47] as adapted by Lyon, Cook, Locke, Ehrhart, and Aarons) measures employees’ shared perceptions of the policies, practices, procedures, and behaviors that are expected, rewarded, and supported in order to facilitate effective EBP implementation. Organizational culture will be measured using the *Organizational Social Context* (OSC [48]) for schools which assesses organizational culture, climate, and work attitudes. The *Implementation Leadership Scale* (ILS; [49] as adapted by Lyon, Cook, Locke, Ehrhart, and Aarons) includes four subscales that assess the degree to which a leader is knowledgeable, supportive, proactive, and perseverant in implementing EBP. Participants will rate the leadership of the person at the next level (e.g., teachers will rate principals, paraprofessionals will rate teachers, trainers and special education directors will rate SELPA leader, principal will rate district leaders). Each leader will also rate themselves. Staff attitudes will be measured using The *evidence-based practice attitude scale* (EBPAS; [40] as adapted by Lyon, Cook, Locke, Ehrhart, and Aarons) that assesses four general attitudes toward adoption of EBIs: appeal, requirements, openness, and divergence*.*

*Resource allocation*. Resources for use of EBP for students with ASD will be measured using the *ASD EBP Resources Assessment Tool* which has been adapted from the *Program Sustainability Tool* v.2; 2013. The survey asks about environmental support from internal (e.g., district leadership) and external (public) sources, funding stability, organizational capacity to implement the practices, program evaluation methods, and program adaptation.

Collaboration and *social network*: Survey participants will report on the top five people they seek out for advice about EBP, their roles and how much their EBP advice network members talk with one another. The egocentric methods we propose for the CAPTAIN study are based on standard industry approaches, used routinely in publications over the past 40 years for several major social science survey work, including the NSF-funded General Social Survey (1985–2014), the National Longitudinal Study of Adolescent to Adult Health (1994–2008), and the National Social Life, Health, and Aging Project (2005–2015).

#### Moderators

*Program size* (measured by the number of students in each SELPA and district), *the proportion of students with ASD in each district*, and *the proportion of students with free and reduced lunch* will be obtained using the most recent data from the California Department of Education. *SELPA structure* will be coded as (1) multi-district; (2) single district; or (3) necessary small SELPA (NSS). The *level of CAPTAIN participation* will be defined by the number of years the SELPA has had a participating cadre member who has met the stated CAPTAIN training goals. *Primary discipline* is defined as the primary educational discipline as reported by the participant.

*ASD experience* will be the reported number of years the participant has been working with students with ASD, in any capacity.

#### Outcomes

The training survey will include information about the *training quality* as rated by the teachers receiving the training. *Training dosage* will include the amount of training time provided for didactic training, coaching (practice with feedback), and ongoing supervision. The *Implementation Citizenship Behavior Checklist* (ICBC; [50] as adapted by Lyon, Cook, Locke, Ehrhart, and Aarons) assesses, via trainer rating, the behaviors educators perform that exceed their expected job tasks to support the implementation of EBP. *EBP dosage* will be measured using the *classroom practice indicator* [51] which has the teacher rate each practice for how often the practice is used during an average school day. The *Survey of Educators Knowledge and Value of Research-Based Practices for Students with Autism* [52] assesses the current level of knowledge and value regarding specific EBP. For each practice, the teachers/paraprofessional participants indicate frequent usage on the *classroom practice indicator* they will receive a fidelity of implementation form from the National Professional Development Center Autism. *Student outcome data* will be gathered through the California Special Education Management Information System (CASEMIS). The 2015–2016 student-level database contains student-level data relevant to this project including demographics, placement type and setting, attendance and behavior reports, and suspensions and expulsions. Data will be collected on attendance, the proportion of educational time the student spends in a general education classroom, number of disciplinary referrals made, number of behavior incident reports filed, and/or the number of days student was suspended or expelled from school.

### Statistical analysis plan

We will begin by examining the independent associations between each malleable factor and the trainer, teacher, and student outcome variables. Based on these results, we will include the factors that have a significant relationship with each outcome in our final models. All models will be hierarchical/multi-level except single-level modeling for outcomes on trainers. More specifically, single-level linear models, binary logistic regression models, multinomial logistic models, and ordinal logistic models (proportional odds models) will be used for modeling continuous, binary, nominal categorical, and ordinal categorical outcomes, respectively, on the trainers. A four-level model will be used for the teacher outcome variables. A five-level model will be used for the student outcome variables. For models with dichotomous outcome variables (i.e., *least restrictive environment*, intensive individual services), a multi-level binary logistic regression model will be used. For all other models, multi-level linear models will be utilized for continuous outcomes. Multi-level multinomial logistic models will be employed for nominal categorical outcomes. Multi-level ordinal logistic models (multi-level proportional odds models) will be used for ordinal categorical outcomes. Moderators will also be considered in the multi-level models as they relate to the outcomes.

### Social network case study mapping the social dynamics of EBP implementation

We will use a dynamic social network approach to map EBP-related connectivity across all identified levels of the system for selected CAPTAIN cadre and their potential collaborators participating the study [53, 54].

Participants: We will select two CAPTAIN cadre members, one high performing and one low performing, based on EBP fidelity and training intensity, from the three SELPA types for a total of six CAPTAIN cadre members. We will then identify and recruit potential collaborators for each of the six CAPTAIN cadre members from the SELPA (*n* = approximately 4 per SELPA), a randomly selected participating district associated with the SELPA (approximately one autism specialist), and two elementary schools (*n* = 10 staff per school) in the selected district for a total of 27 people per type of SELPA, for an overall total of about 81 people.

Social network measures: We will adapt a previously pilot-tested approach used to map the *social dynamics of intervention* (SoDI). The SoDI was successfully developed and piloted by McGhee Hassrick and colleagues [53, 54] in a previous NIH funded study (R21HD067865-01). The SoDI uses traditional social network analytics to map multiplex connectivity. The adapted SoDI will measure the density of three different person-to-person networks among people at the SELPA, district, school and classroom levels who are supporting and providing EBP intervention for children with ASD. Using descriptive analysis, we will compare the social networks of the high-performing cadres with those of the low-performing cadres, to determine how network configuration varies.

*Data Analysis.* We will use ORA software for dynamic network analysis [55] which relies on traditional social network measures to inventory EBP advice, problem-solving and trust networks [56], and dynamic network measures including EBP training, resources, and intervention use among school staff [55] to calculate patterns of alignment, using EBP exclusivity and EBP expertise measures. Once we have taken an inventory of EBP training, resources, and intervention activities, the impact that the CAPTAIN cadre has had on classroom practices can be more thoroughly assessed. These multi-nodal and multiplex inventories will help us to measure the system of EBP activities among school staff and their district and state partners. We can gauge the amount of social interaction people have with one another, and also the degree and ways EBP trainings, resources, and interventions are shared or not shared, among school staff, including teachers, paraprofessionals, and principals.

## Discussion

Identifying malleable factors that influence the implementation of EBP across system levels for students with ASD will help policymakers and administrators make system-wide changes to positively impact teacher training and effective use of EBP both for ASD and more generally. Characterization of malleable organizational factors with accompanying linkage to implementation and clinical outcomes is an innovative process with the potential to increase understanding of the mechanisms of action of system level implementation factors. The CAPTAIN statewide implementation effort provides a natural vehicle for examining facilitators and barriers to training and support for community providers. Outcomes will inform scale-up efforts for EBP implementation broadly and implementation science methods.

In addition, to our knowledge, no one has used formal social network analysis to capture multi-level collaboration among state-, district-, school-, and classroom-level stakeholders in the field of ASD. The proposed research will advance our scientific understanding of social determinants of treatment and outcomes for children with ASD and provide new conceptual tools for understanding disparities in treatment and outcomes. The proposed study both compliments and redirects existing research by considering the configuration of the social networks of EBP supporters and providers across system levels, overcoming previous methodological barriers that constrain service research to only consider one level per study. In addition to new approaches in research, the proposed project provides the basis for new social network interventions in autism that strengthen the capacity of providers to work together to provide higher quality EBP for children with ASD.

Findings will add to the very limited research examining cross-level mechanisms linking both inner and outer context factors to changes in provider behaviors [31] and to consumer outcomes. Data from this exploratory project will be used to develop an implementation assessment intervention package that will address malleable factors identified as important to the teacher and student outcomes. This project may provide a generalizable model for the development of multi-level implementation interventions across complex service settings.

## Abbreviations

ASD: Autism spectrum disorder
CAPTAIN: California Autism Professional Training and Information Network
EBP: Evidence-based practice
EPIS: Exploration, Preparation, Implementation, and Sustainment
