# Correction to: Exploring multi-level system factors facilitating educator training and implementation of evidence-based practices (EBP): a study protocol

**DOI:** 10.1186/s13012-018-0750-9

**Published:** 2018-04-23

**Authors:** Aubyn C. Stahmer, Jessica Suhrheinrich, Patricia L. Schetter, Elizabeth McGhee Hassrick

**Affiliations:** 1 University of California, Davis MIND Institute, 2825 50th St, Sacramento, CA 95819 USA; 2 College of Education, San Diego State University, 5500 Campanile Dr., San Diego, CA 92182 USA; 3 Department of Psychiatry and Behavioral Sciences, University of California, Davis, Sacramento, USA; 4 Center of Excellence in Developmental Disabilities, University of California, Davis, Sacramento, USA; 5 Child and Adolescent Services Research Center, San Diego, USA; 6 Life Course Outcomes Research Program at AJ Drexel Autism Institute, Drexel University, 3020 Market St, St 560, Philadelphia, PA 19104 USA

## Correction

After publication of the original article [1] it was brought to our attention that author Elizabeth McGhee Hassrick was erroneously included as Elizabeth McGee Hassrick. The correct spelling of the author’s name is included in the author list of this Correction.
